# A Pleiotrophin C-terminus peptide induces anti-cancer effects through RPTPβ/ζ

**DOI:** 10.1186/1476-4598-9-224

**Published:** 2010-08-25

**Authors:** Zoi Diamantopoulou, Oya Bermek, Apostolos Polykratis, Yamina Hamma-Kourbali, Jean Delbé, José Courty, Panagiotis Katsoris

**Affiliations:** 1 Division of Genetics, Cell and Developmental Biology, Department of Biology, University of Patras, Greece; 2 Laboratoire de recherche sur la Croissance Cellulaire, la Réparation et la Régénération Tissulaires (CRRET), CNRS UMR 7149, Université Paris XII, Avenue du Général de Gaulle, 94010 Créteil Cedex, France

## Abstract

**Background:**

Pleiotrophin, also known as HARP (Heparin Affin Regulatory Peptide) is a growth factor expressed in various tissues and cell lines. Pleiotrophin participates in multiple biological actions including the induction of cellular proliferation, migration and angiogenesis, and is involved in carcinogenesis. Recently, we identified and characterized several pleiotrophin proteolytic fragments with biological activities similar or opposite to that of pleiotrophin. Here, we investigated the biological actions of P(122-131), a synthetic peptide corresponding to the carboxy terminal region of this growth factor.

**Results:**

Our results show that P(122-131) inhibits *in vitro *adhesion, anchorage-independent proliferation, and migration of DU145 and LNCaP cells, which express pleiotrophin and its receptor RPTPβ/ζ. In addition, P(122-131) inhibits angiogenesis *in vivo*, as determined by the chicken embryo CAM assay. Investigation of the transduction mechanisms revealed that P(122-131) reduces the phosphorylation levels of Src, Pten, Fak, and Erk^1^/_2_. Finally, P(122-131) not only interacts with RPTPβ/ζ, but also interferes with other pleiotrophin receptors, as demonstrated by selective knockdown of pleiotrophin or RPTPβ/ζ expression with the RNAi technology.

**Conclusions:**

In conclusion, our results demonstrate that P(122-131) inhibits biological activities that are related to the induction of a transformed phenotype in PCa cells, by interacing with RPTPβ/ζ and interfering with other pleiotrophin receptors. Cumulatively, these results indicate that P(122-131) may be a potential anticancer agent, and they warrant further study of this peptide.

## Background

Pleiotrophin, also known as HARP (Heparin Affin Regulatory Peptide) is a 136-amino acid, secreted growth factor that, along with Midkine, constitutes a two-member sub family of heparin binding growth factors (HBGFs). Although pleiotrophin has been shown to promote neurite outgrowth in the developing brain [1], elevated concentrations of this growth factor are found in many types of tumors as well as in the plasma of patients with different types of cancer [2-4]. Pleiotrophin induces a transformed phenotype in several cell lines [5,6] and exhibits mitogenic, anti apoptotic, chemotactic, and angiogenic actions *in vitro *as well as *in vivo *[7-10].

The biological activities of pleiotrophin are mediated by three distinct receptors: SDC3 (N-Syndecan) [11], Receptor Protein Tyrosine Phosphatase (RPTPβ/ζ) [12], and Anaplastic Lymphoma Kinase (ALK) [13]. N-Syndecan and RPTPβ/ζ have been implicated in neurite outgrowth [10,11], while RPTPβ/ζ and ALK have been shown to mediate cellular migration induced by pleiotrophin as well as the mitogenic, angiogenic, and transforming activities of this growth factor [14-18].

Growth factors can be hydrolyzed by proteases, leading to the production of biological active peptides. Previous studies indicate that pleiotrophin is cleaved by enzymes in the extracellular environment, such as plasmin, trypsin, chymotrypsin, and MMPs. Moreover, the resulting peptides exert altered biological functions compared to the whole molecule. The proteolytic cleavage of pleiotrophin is also affected by the presence of glycosaminoglycans (GAGs), suggesting that a complex system serves to regulate the overall effect of this growth factor [19,20]. Furthermore, pleiotrophin and pleiotrophin peptides modulate the biological actions of other growth factors such as VEGF, contributing to the complex mode of growth factor actions [21].

Prostate cancer (PCa) is the most common cancer among men in Western countries, although the development of PCa as well as the signals contributing to the transformed phenotype of PCa cells remains incompletely understood [22]. During adulthood, maintenance of normal prostate function depends on mesenchymal-epithelial interactions, which contribute to the homeostatic equilibrium of the glandular prostate epithelial cells. Disturbances in this equilibrium lead to the development of diseases like PCa. Although the mechanisms that control the mesenchymal-epithelial interactions are poorly understood, numerous studies suggest that growth factors have a key role in prostate homeostasis. Pleiotrophin has been implicated in PCa progression and acts as an autocrine growth factor in various prostate-derived cell lines including DU145, PC3, and LNCaP [23,24].

Truncated forms of pleiotrophin or synthetic peptides corresponding to defined domains of this growth factor have been studied in an attempt to understand the structure/function relationship of pleiotrophin [25-27]. We previously reported that the biological effects of this growth factor were inhibited by the truncated mutant PTNΔ111-136 and corresponding synthetic peptide P(111-136) [28]. In the context of defining peptides with anti tumor actions, we sought to identify the minimum sequence responsible for the inhibition of pleiotrophin activity. Since an obvious feature of P(111-136) is the stretch of basic residues, we investigated whether the basic sequence P(122-131) (KKKKKEGKKQ) may have biological activities that are related to the induction of a transformed phenotype in PCa cells. Here, we investigated the effect of P(122-131) on the adhesion, proliferation, and migration of two prostate epithelial cell lines as well as on *in vivo *angiogenesis.

## Results

In a previous work, we reported that P(122-131) inhibits anchorage-independent growth of DU145 prostate cancer cells [29]. In the present work, we tested the effect of P(122-131) on other tumor phenotypes in the well-established prostate carcinoma cell lines, DU145 and LNCaP. We also investigated the effect of these peptides on angiogenesis *in vivo*, using the CAM assay. Since P(122-131) contains seven lysines and is highly charged, we also examined the effects of two "mock" peptides in parallel. One consisted of D-amino acids (designated AAD), while the other consisted of five lysines (designated 5K).

### P(122-131) inhibits adhesion of DU145 and LNCaP cells

**Figure 1:**
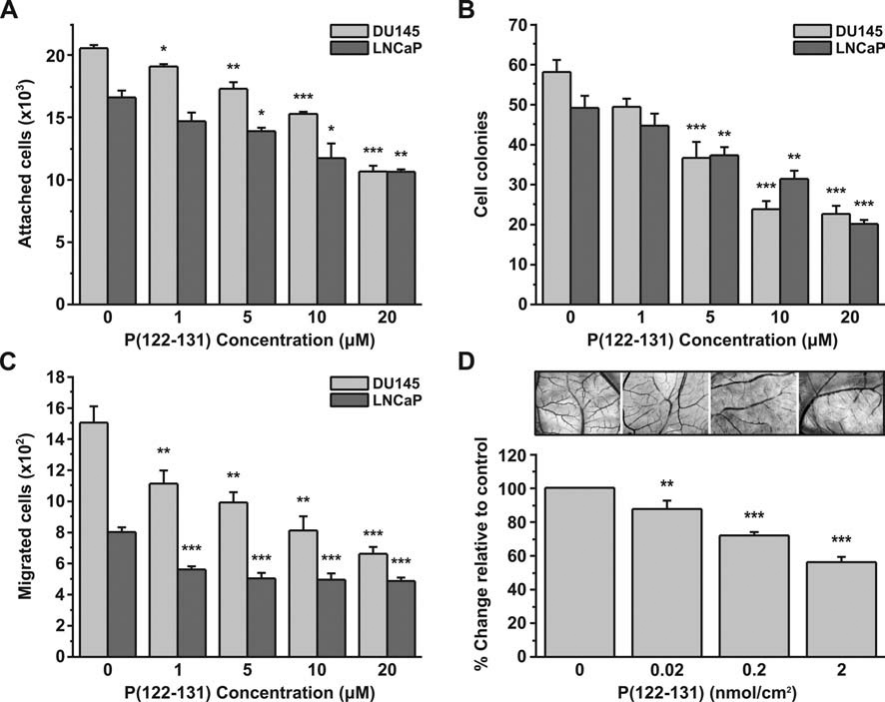
**P(122-131) promotes anti-tumor phenotypes in DU145 and LNCaP cells and inhibits angiogenesis *in vivo***. (A) Number of adherent DU145 and LNCaP cells in the presence of increasing concentrations of P(122-131). An equal number of DU145 or LNCaP cells was incubated with increasing concentrations of P(122-131) for 30 min before seeding. After a 10-min incubation period, adherent cells were measured by the crystal violet assay. (B) Soft agar growth assays showing anchorage-independent proliferation. An equal number of DU145 or LNCaP cells was resuspended in growth medium containing 10% FBS, 0.3% agar, and increasing concentrations of P(122-131), and seeded onto the bottom agar, which consisted of growth medium containing 10% FBS and 0.8% agar. The top agar was allowed to solidify, and standard growth media supplemented with peptide was added to each well. The cells were incubated 12 days, after which cell colonies larger than 50 μm were quantified by counting the entire area of each well. (C) Migration of cells through Transwell filters. The lower compartment of Transwell filters (8 μm pores) was filled with growth media containing 2.5% FBS, 0.5% BSA, and increasing concentrations of P(122-131). An equal number of DU145 or LNCaP cells was resuspended in growth medium containing 2.5% FBS and 0.5% BSA, and transferred into Transwell inserts. Cells that successfully migrated through the filter pores, were fixed, stained and quantified by counting the entire area of each filter. (D) Effect of P(122-131) on angiogenesis, as measured by the chicken embryo CAM assay. An 1 cm^2 ^area of chicken embryo CAM, restricted by a silicon ring, was incubated with increasing concentrations of P(122-131). 48 h later total vessels length was quantified as described in Materials and Methods. Results are mean values ± SE from at least 3 independent experiments.

The effect of P(122-131) on the adhesion of DU145 cells was tested using three approaches. First, an equal number of cells was incubated with increasing concentrations of peptides and immediately seeded. In the second approach, cells were incubated with different concentrations of the peptides for 30 min before seeding. In the final approach, cells were pre-incubated for 30 min with increasing concentrations of peptide, then washed and seeded. After a 10-min incubation period, adherent cells were measured by the crystal violet assay. Under all conditions, P(122-131) inhibited adhesion in a concentration dependent manner, having a maximal effect (50% inhibition relative to control) at a concentration of 20 μΜ (Figure 1A). To confirm this result, we looked for inhibitory effect of P(122-131) on the adhesion of LNCaP cells. As shown in Figure 1A, P(122-131) inhibited LNCaP adhesion in a concentration-dependent manner, having a maximal effect (40% inhibition relative to control) at a concentration of 20 μΜ.

### P(122-131) inhibits anchorage-independent proliferation of DU145 and LNCaP cells

The effect of P(122-131) on the proliferation of DU145 and LNCaP cells was investigated. We found that P(122-131) inhibited anchorage-independent proliferation in a concentration-dependent manner, having a maximal effect (60% inhibition relative to control) at a concentration of 20 μΜ (Figure 1B).

### P(122-131) inhibits migration of DU145 and LNCaP cells

We next investigated the effect of P(122-131) on DU145 and LNCaP chemotaxis, as measured using Transwell assays. Similar to its effects on adhesion and proliferation, P(122-131) inhibited chemotactic migration in a concentration-dependent manner (55% inhibition relative to control). The maximal effect was observed at the concentration of 20 μM (Figure 1C).

### P(122-131) inhibits the *in vivo *angiogenesis

The inhibitory effects of P(122-131) on DU145 and LNCaP adhesion, proliferation, and migration are consistent with a possible anti-tumor action for this peptide. Therefore, we tested the effect of this peptide on *in vivo *angiogenesis. Tumor angiogenesis plays a key role in cell proliferation by providing nutrients and oxygen. It also facilitates metastasis through the formation of new, leaky vessels. We observed that P(122-131) reduced the total length of blood vessels in the CAM assay in a concentration-dependent manner. Angiogenesis was inhibited up to 45%, with maximal inhibition occurring in the presence of 2 nmol P(122-131) (Figure 1D).

**Figure 2:**
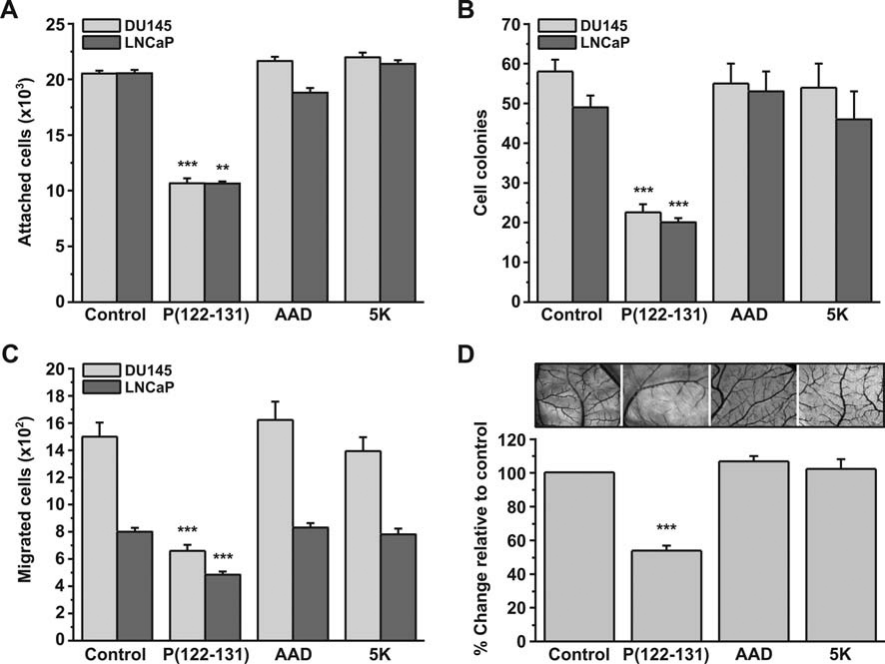
**P(122-131) inhibitory effect is attributed to its specific amino acid sequence and charge**. (A) Number of adherent DU145 and LNCaP cells in the presence of 20 μM P(122-131), AAD, or 5K, as determined by the crystal violet assay. (B) Soft agar growth assays showing anchorage-independent proliferation. (C) Migration of cells through Transwell filters. (D) Effect of 2 nmol P(122-131), AAD, or 5K on angiogenesis as measured by the chicken embryo CAM assay. Results are mean values ± SE from at least 3 independent experiments.

In contrast to P(122-131), neither AAD nor 5K had any measurable effect on angiogenesis. Similarly, neither affected adhesion, proliferation, nor migration of DU145 or LNCaP cells (Figure 2).

### P(122-131) binds to RPTPβ/ζ and is endocytosed

**Figure 3:**
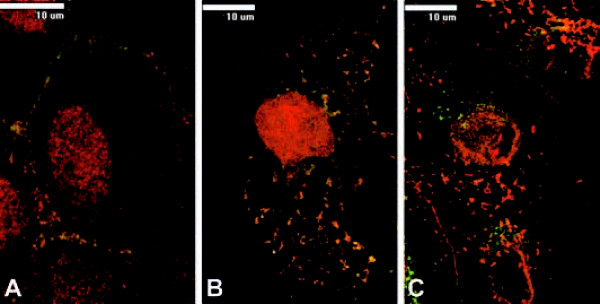
**P(122-131) binds to RPTPβ/ζ**. Merged images of overlapping co-labelling for biotinylated P(122-131), [B(122-131)] (green) and RPTPβ/ζ (red). Incubation of DU145 cells with B(122-131) for 30 sec (A) or for 20 min (B). Incubation of DU145 cells with B(122-131) and pleiotrophin for 20 min (C). Overlapping labelling appears yellow. B(122-131) was visualized using streptavidin-FITC, while RPTPβ/ζ using specific monoclonal antibody and rhodamine conjugated secondary antibody.

P(122-131) harbours a cluster of basic residues known to bind to cell receptors [25]. To begin to understand the mechanism through which P(122-131) exerts its biological actions, we investigated the effect of this peptide on signaling mediated by the pleiotrophin receptors. In a previous work, co-immunoprecipitation/Western blot analysis of P(122-131) and RPTPβ/ζ indicated that this pleiotrophin receptor interacts with P(122-131) [29]. To provide additional support for an interaction between P(122-131) and RPTPβ/ζ, DU145 cells were co-labelled for B(122-131) and RPTPβ/ζ. After a 30-sec incubation of cells with B(122-131), confocal microscopy revealed B(122-131) bound to the cell surface and co-localized with RPTPβ/ζ (Figure 3A). After a 20-min incubation, endocytotic vesicles containing both B(122-131) and RPTPβ/ζ were detected in the cytoplasm (Figure 3B). Furthermore, as shown in Figure 3C, P(122-131) association with RPTPβ/ζ was displaced by pleiotrophin. As expected, incubation of cells with only streptavidin-FITC or rhodamine conjugated secondary antibodies produced no signal (data not shown).

### Elucidation of the mechanism through which P(122-131) exerts its biological actions

DU145 and LNCaP cells synthesize and secrete pleiotrophin, which in an autocrine manner stimulates cells [3,24]. To examine whether the inhibitory effects of P(122-131) on DU145 adhesion, proliferation, and migration may be the result of endogenous pleiotrophin inhibition, we stably transfected DU145 cells with pcDNA3.1+ plasmid encoded the antisense mRNA of pleiotrophin. After 1 month of selection with neomycin, clones were screened for down-regulation of pleiotrophin expression. Strong down-regulation of pleiotrophin expression was observed in clones #2 and #5 (DU145-HM2 and DU145-HM5 respectively). No reduction of the pleiotrophin expression was observed in cells transfected with pcDNA3.1+ alone (DU145-NC1, DU145-NC3) (Additional file 1-A).

**Figure 4:**
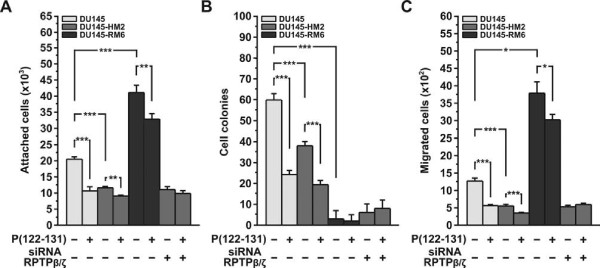
**Elucidation of the mechanism through which P(122-131) exerts its biological actions**. Effect of P(122-131) on adhesion (A), anchorage-independent proliferation (B), and migration (C) of DU145, DU145-HM2, and DU145-RM6 cells. DU145-HM2 cells transfected with pleiotrophin antisense RNA, DU145-RM6 cells transfected with shRNA targeting RPTPβ/ζ. The last two bars of each diagram indicate DU145-HM2 cells that were transiently transfected with siRNA targeting RPTPβ/ζ. Results are mean values ± SE from at least 3 independent experiments.

As shown in Figure 4A, pleiotrophin knockdown decreased DU145 adhesion, and P(122-131) further decreased it. However, the inhibitory effect of P(122-131) on the adhesion of DU145-HM2 cells was up to 20%, while its inhibitory effect on DU145 adhesion was up to 50%. No difference between the adhesion of DU145 and DU145-NC3 cells was observed (data not shown). These results indicate that P(122-131) not only inhibits pleiotrophin-mediated adhesion, but also may interfere with other growth factors actions. Furthermore, since pleiotrophin enforces dimerization of RPTPβ/ζ, inactivates its catalytic activity [30], and inhibits cellular adhesion (unpublished data), the inhibitory effect of P(122-131) may also be the result of its interaction with RPTPβ/ζ. To examine whether RPTPβ/ζ mediates the inhibitory effect of P(122-131), we tested the effect of P(122-131) on DU145 cells with stable down-regulation of RPTPβ/ζ expression (DU145-RM6). We found that P(122-131) inhibits DU145-RM6 adhesion up to 20% (Figure 4A), indicating that the peptide interferes with other pleiotrophin receptors or with other growth factors. To determine whether P(122-131) may interfere with other growth factors, we transiently transfected DU145-HM2 cells with a siRNA targeting the mRNA of RPTPβ/ζ. In parallel, DU145-HM2 cells were transiently transfected with a siRNA that does not target any mRNA (negative control) (Additional file 1-B). As shown in Figure 4A, pleiotrophin/RPTPβ/ζ knockdown decreased DU145 adhesion, and blocked the inhibitory effect of P(122-131). No difference between the adhesion of DU145-HM2 and DU145-HM2 cells transfected with the negative control siRNA was observed (data not shown). Taken together, these results indicate that P(122-131) interacts with RPTPβ/ζ and induces RPTPβ/ζ inhibitory effect on cellular adhesion, while in parallel antagonizes the interaction of pleiotrophin with its other receptors, probably SDC3, inhibiting pleiotrophin-induced adhesion.

As shown in Figure 4B, pleiotrophin knockdown decreased DU145 anchorage-independent proliferation, and P(122-131) further decreased it. However, the inhibitory effect of P(122-131) on this biological action was not as effective as on wild type cells. These results indicate that P(122-131) interferes with pleiotrophin and other growth factors actions. However, the effect of RPTPβ/ζ knockdown on the formation of cell colonies was so strong that we cannot draw any conclusion about the mechanism that P(122-131) inhibits anchorage-independent proliferation.

As shown in Figure 4C, pleiotrophin knockdown decreased DU145 chemotactic migration, and P(122-131) further decreased it. However, the inhibitory effect of P(122-131) on the migration of DU145-HM2 cells was up to 20%, while its inhibitory effect on DU145 migration was up to 55%. No difference between the migration of DU145 and DU145-NC3 cells was observed (data not shown). These results indicate that P(122-131) not only inhibits pleiotrophin-mediated migration, but also may interfere with other growth factors signaling. Furthermore, since pleiotrophin enforces dimerization of RPTPβ/ζ, inactivates its catalytic activity [30], and inhibits cellular migration (unpublished data), the inhibitory effect of P(122-131) may also be the result of its interaction with RPTPβ/ζ. To examine whether RPTPβ/ζ mediates the inhibitory effect of P(122-131), we tested the effect of P(122-131) on DU145-RM6 cells. We found that P(122-131) inhibits DU145-RM6 migration up to 20% (Figure 4C), indicating that the peptide interferes with other pleiotrophin receptors or with other growth factors. To determine whether P(122-131) may interfere with other growth factors, we examined its effect on cells in which both pleiotrophin and RPTPβ/ζ expression was down-regulated. As shown in Figure 4C, pleiotrophin/RPTPβ/ζ knockdown decreased DU145 migration, and blocked the inhibitory effect of P(122-131). No difference between the migration of DU145-HM2 and DU145-HM2 cells transfected with the negative control siRNA was observed (data not shown). Taken together, these results indicate that P(122-131) interacts with RPTPβ/ζ and induces RPTPβ/ζ inhibitory effect on cellular migration, while in parallel antagonizes the interaction of pleiotrophin with its others receptors, probably SDC3, inhibiting pleiotrophin-induced migration.

### P(122-131) inactivates Src, Fak, and Erk^1^/_2_, and activates Pten

**Figure 5:**
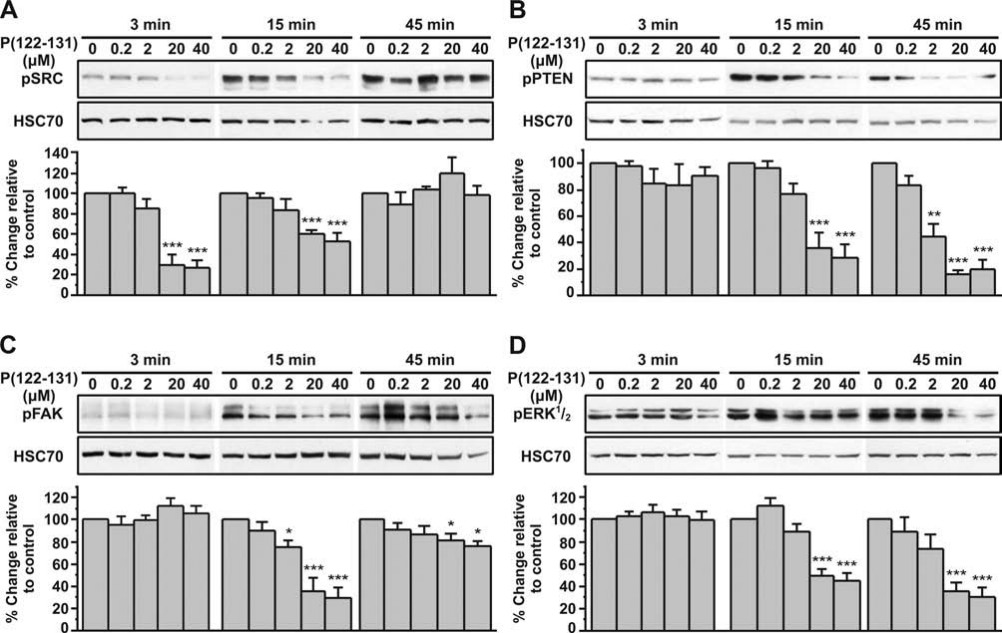
**Src, Fak, and Erk**^**1**^**/**_**2 **_**are inactivated by P(122-131), while PTEN is activated**. Western blot analysis of phosphorylated Src(A), Fak (B) Pten (C), and Erk^1^/_2 _(D), in cells stimulated with increasing concentrations of P(122-131) for 3, 15, and 45 minutes. The blots were stripped and reprobed for HSC70. Results are expressed as % change relative to control and are mean values ± SE from at least 3 independent experiments.

Src activation is strictly regulated and depends on dephosphorylation of Y527 in the carboxy-terminal tail, which is prerequisite for its subsequent activation by autophosphorylation of Y416 in the activation loop of the kinase [31]. To determine whether Src may be affected by P(122-131), DU145 cells were serum starved for 4 h, then incubated with increasing concentrations of P(122-131) for 3 to 45 min. Src inactivation was indirectly assessed by Western blot analysis of phosphorylated Src at site Y416 and HSC70 that was used to normalize the results. As shown in Figure 5A, P(122-131) promoted a swift decrease in Src phosphorylation within 3 min in a concentration dependent manner, having a maximal effect (70% inhibition relative to control) at a concentration of 20 μΜ. This inactivation returned to near basal levels by 45 min (Figure 5A).

We next investigated the effects of P(122-131) on inactivation of other molecules known to interact with Src. We found that FAK phosphorylation was decreased 15 min after incubation of DU145 cells with P(122-131) in a concentration dependent manner, having a maximal effect (70% inhibition relative to control) at a concentration of 20 μΜ. This inactivation returned to near basal levels by 45 min (Figure 5C). As shown in Figure 5D, ERK^1^/_2 _were also inactivated after a 15-min incubation with P(122-131) that was sustained up to 45 min (70% inhibition relative to control). Finally, PTEN was activated after a 15-min incubation with P(122-131) that was sustained up to 45 min (80% inhibition relative to control) (Figure 5B).

### Elucidation of the mechanism through which P(122-131) affects Src, Fak, Pten, and Erk^1^/_2 _activity

**Figure 6:**
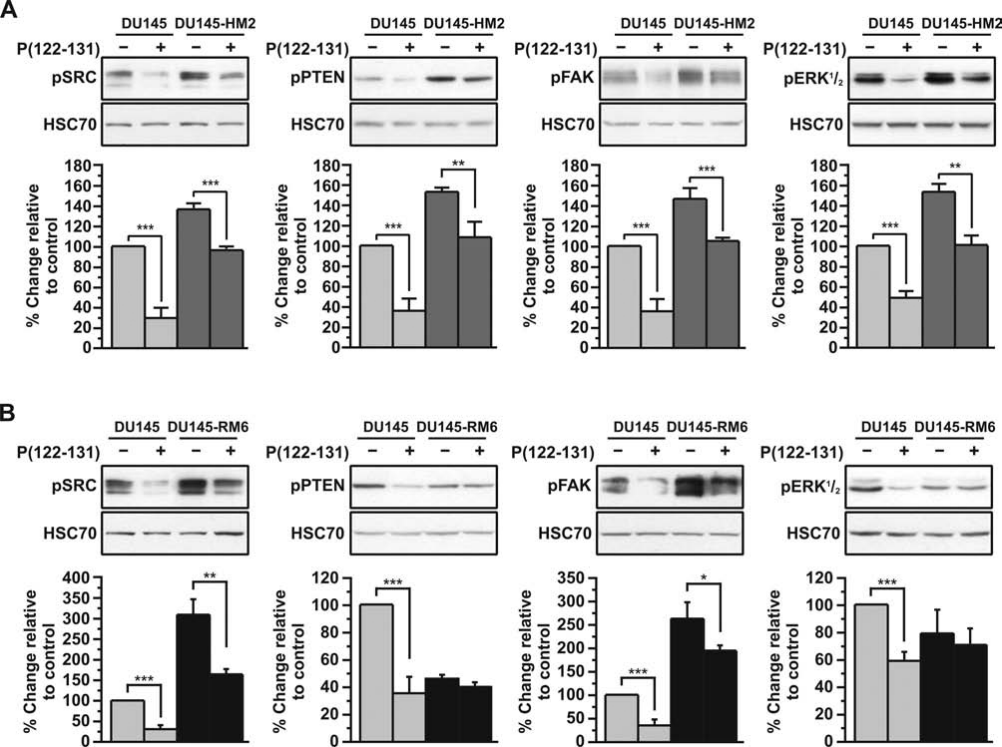
**Effect of pleiotrophin or RPTPβ/ζ knockdown on P(122-131)-induced kinase signaling**. Western blot analysis of phosphorylated Src, Fak, Pten, and Erk^1^/_2 _in DU145-HM2 (A) and DU145-RM6 cells (B) stimulated with 20 μM P(122-131) for 3 minutes (Src) or 15 minutes (Fak, Pten, and Erk^1^/_2_). The blots were stripped and reprobed for HSC70. Results are expressed as% change relative to control and are mean values ± SE from at least 3 independent experiments.

In a previous study, we have shown that pleiotrophin enforces RPTPβ/ζ dimerization and inactivation, and reduces the phosphorylation levels of Src, Fak, Pten, and Erk^1^/_2 _(unpublished data). In this study, we showed that P(122-131) interacts with RPTPβ/ζ and induces RPTPβ/ζ inhibitory effect on cellular adhesion and migration, while in parallel antagonizes the interaction of pleiotrophin with its others receptors, probably SDC3, inhibiting pleiotrophin-induced biological actions. To confirm that P(122-131) interferes with pleiotrophin signaling, we tested its effect on activation of Src, Fak, Pten, and ERK^1^/_2 _of DU145-HM2 cells. As shown in Figure 6A, the phosphorylation levels of Src, Fak, Pten, and Erk^1^/_2 _are induced on DU145-HM2 cells compared with wild type cells, while pleiotrophin knockdown partially blocked P(122-131)-induced Src, Fak and Erk^1^/_2 _inactivation, and Pten activation. Since pleiotrophin expression levels are low on DU145-HM2 cells, RPTPβ/ζ cannot be dimerized, and as monomer reduces the phosphorylation of Src at site Y527 and induces autophosphorylation of Y416, resulting in increased Fak, Pten, and Erk^1^/_2 _phosphorylation. However, when DU145-HM2 cells are treated with P(122-131), it cannot inhibit pleiotrophin signaling, but can reduce the phosphorylation levels of Src, Fak, Pten, and Erk^1^/_2 _through RPTPβ/ζ. Furthermore, as shown in Figure 6B, RTPPβ/ζ knockdown partially blocked P(122-131)-mediated Src and Fak inactivation, and inhibited P(122-131)-mediated Pten activation and Erk^1^/_2 _inactivation. Taken together, these results indicate that P(122-131)/RPTPβ/ζ interaction triggers a signal transduction pathway that reduces the phosphorylation levels of these four signal transduction molecules, and inhibits cellular adhesion and migration, while in parallel P(122-131) interferes with other pleiotrophin receptors, probably SDC3, inhibits pleiotrophin-mediated Src and Fak activation, resulting in inhibition of pleiotrophin-induced cellular adhesion and migration.

## Discussion

During the last decade, pleiotrophin has come to be recognized as a pleiotropic growth factor that participates not only in neurite outgrowth in the developing brain [1], but also in angiogenesis, and malignant transformation of many cell types. Pleiotrophin is elevated in sera or tumors from patients with colon, stomach, pancreatic, and breast cancer [2-10]. Moreover, the differential expression of pleiotrophin mRNA and protein among normal and malignant prostate epithelial cells, implicates this protein in the induction of a transformed phenotype [24].

NMR studies showed that pleiotrophin contains two β-sheet domains connected by a flexible linker. In addition, its two lysine cluster sequences within both the N- and C-terminal domains lack a detectable structure and appear to form random coils [32]. To date, pleiotrophin activities have been attributed either to the entire molecule or to specific domains. From previous studies, it is known that either but not both the N- or C-terminal domains is required for pleiotrophin activity [27], and that the C-terminal domain is involved in the mitogenic, angiogenic, and tumor formation activities of this growth factor [25,28]. Furthermore, pleiotrophin peptide fragments have been detected in cell supernatants, as well as in tissues [33,34], and such peptides can also be generated *in vitro *by proteolytic cleavage of pleiotrophin [20]. Our group has already characterized the biological actions of several pleiotrophin peptides [10,20,25,28]. It is noteworthy, that although pleiotrophin N- and C-terminal domains lack a detectable structure, peptides corresponding to these domains induce *in vitro *and *in vivo *angiogenesis [10,33]. Therefore, the biological actions of pleiotrophin should be always considered to be the overall outcome of its secretion, degradation, and specific cleavage, with latter event possibly generating pleiotrophin peptides with diverse, or even opposite, biological actions. To illustrate this point, a study on glioblastoma cell proliferation and migration has revealed that cleavage of the 12 C-terminal amino acids from pleiotrophin (124-136) leads to distinct biological activities through differential activation of RPTPβ/ζ or ALK signalling pathways [15].

In this study, we sought to identify the minimum sequence of the C-terminal region of pleiotrophin that is responsible for the inhibition of biological activities that are related to the induction of a transformed phenotype in PCa cells. Since an obvious feature of pleiotrophin C-terminal domain is the stretch of basic residues, we investigated the effect of the basic sequence P(122-131) (KKKKKEGKKQ) on tumor phenotypes. Our results showed that P(122-131) inhibits DU145 and LNCaP cell adhesion, anchorage-independent proliferation, and migration in a concentration dependent manner. Furthermore, the CAM assay revealed that P(122-131) suppressed the formation of new blood vessels, a process important for tumor growth and metastasis. These biological activities of P(122-131) could be attributed solely to its high positive charge. Nevertheless, this does not seem to be the case, since, in the same set of experiments, neither AAD nor 5K exerted any detectable biological activity. Thus, the action of P(122-131) is more likely due to its specific amino acid sequence and charge.

To reveal the mechanism through which P(122-131) exerts its biological actions, we investigated the effect of this peptide on signaling mediated by the pleiotrophin receptors. Pleiotrophin binds to specific cell surface receptors such as SDC3 [11], ALK [13], and RPTPβ/ζ [12]. RPTPβ/ζ is synthesized as a membrane-bound CS proteoglycan and its extracellular variant, which is generated by alternative splicing, is phosphacan, a major soluble CS proteoglycan [12,35]. Pleiotrophin binding to RPTPβ/ζ depends on the CS portion of this receptor, and the removal of CS results in a remarkable decrease in binding affinity [36]. However, treatment of cells with chondroitinase had no effect on the binding of P(122-131) to DU145 cells, suggesting that P(122-131) does not bind to the RPTPβ/ζ-derived glycosaminoglycans, in spite of its basicity [29]. Our results demonstrate that P(122-131) actions are mediated by RPTPβ/ζ. P(122-131) was co localized with RPTPβ/ζ at the cell surface and eventually become cytoplasmic, likely as a result of endocytosis. Moreover, immunoprecipitation followed by Western blotting confirms the interaction between P(122-131) and RPTPβ/ζ [29].

RPTPβ/ζ is a receptor phosphatase with intrinsic catalytic activity [30]. In a previous study, we showed that pleiotrophin/RPTPβ/ζ interaction leads to different biological responses according to RPTPβ/ζ substrates. Pleiotrophin/RPTPβ/ζ-Src interaction reduces the phosphorylation levels of Src, Fak, Pten, and Erk^1^/_2_, and inhibits cellular adhesion and migration (unpublished data). Investigation of the transduction mechanism revealed that P(122-131) induced Src, Fak, and Erk^1^/_2 _inactivation in a concentration and time-dependent manner. Furthermore, P(122-131) activated Pten, a tumor suppressor which activity has been proposed to reduce cell migration and proliferation [37,38].

The finding that the inhibitory effect of P(122-131) on cellular adhesion and migration could be reduced by down-regulation of pleiotrophin or RPTPβ/ζ expression, demonstrates that this peptide not only ineracts with RPTPβ/ζ and inhibits cellular adhesion and migration, but also antagonizes the interaction of pleiotrophin with its others receptors. P(122-131) interference with other pleiotrophin receptors was confirmed by the finding that P(122-131) induced Src and Fak inactivation on cells with RPTPβ/ζ knockdown. Furthermore, we excluded the possibility of P(122-131) interference with other growth factors, since the peptide did not exert any biological action on cells that the expression levels of both pleiotrophin and RPTPβ/ζ are down-regulated.

Our results also showed that P(122-131) inhibits anchorage-independent proliferation, but the effect of RPTPβ/ζ knockdown was so strong that we cannot draw any conclusion about the mechanism through which the peptide inhibits this biological action. It is known that RPTPs show structural and functional similarity to CAMs. Although certain RPTPs mediate homophilic interactions [39], there are no data indicating that RPTPβ/ζ is implicated in such interactions.

## Conclusions

**Figure 7:**
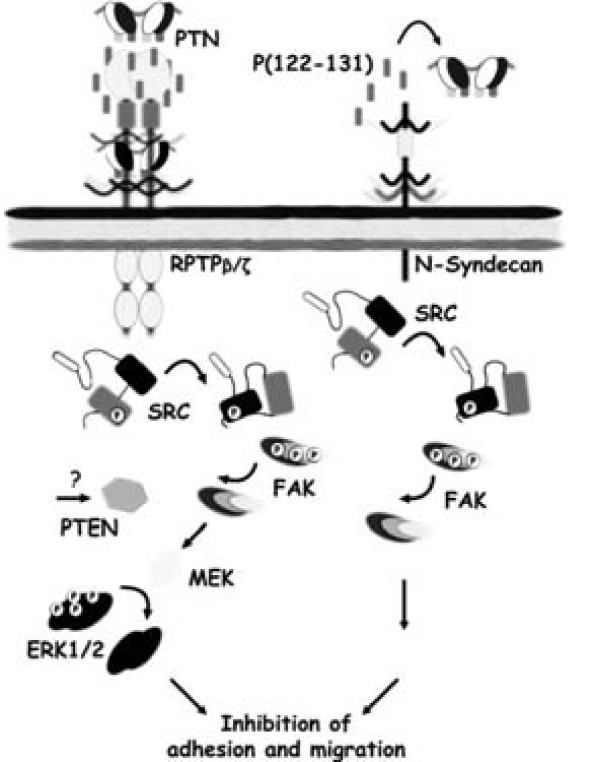
**Suggested mechanism of P(122-131) action on DU145 cells that express only RPTPβ/ζ and N-Syndecan**.

In the context of studying the functions of specific domains of pleiotrophin and defining peptides with anti tumor actions, we identified the minimum sequence responsible for the inhibition of pleiotrophin activity. Our results demonstrated that P(122-131) interacts with RPTPβ/ζ and triggers a signal transduction pathway that inhibits DU145 and LNCaP adhesion and migration, while in parallel antagonizes the interaction of pleiotrophin with its others receptors, inhibiting pleiotrophin-induced biological actions (Figure 7). Cumulatively, these results indicate that P(122-131) may be a potential anticancer agent, and they warrant further study of this peptide.

## Methods

### Materials

Standard Boc amino acids, p-methylbenzhydrylamine-polystyrene resin (0.81 mmol NH2/g), and O-(benzotriazol-1-yl)-1,1,3,3-tetramethyluronium hexafluorophosphate (HBTU) were purchased from Senn Chemicals. Solvents (peptide synthesis grade) and other reagents were obtained from Applied Biosystems. Cell culture reagents were from BiochromKG (Seromed, Germany). All other reagents were purchased from Sigma-Aldrich.

Polyclonal antibodies against pSrc (Tyr416), pFak (Tyr925), pPten (Ser380), pAkt (Ser473), and pErk^1^/_2 _(Thr202/Tyr204), as well as monoclonal antibodies against Src (36D10) were purchased from Cell Signaling Technology. Polyclonal antibodies against HSC70 were purchased from Santa Cruz Biotechnology, Inc. Monoclonal anti-RPTPβ/ζ antibodies were from BD Transduction Laboratories (San Diego, CA), and actin polyclonal antibody was purchased from Sigma-Aldrich.

### Cell culture

The human prostate cancer epithelial cell lines DU145 and LNCaP (ATCC) were grown in RPMI-1640 medium supplemented with 10% fetal bovine serum (FBS), 100 U/ml penicillin, and 100 μg/ml streptomycin. Cultures were maintained in 5% CO_2 _and 100% humidity at 37°C.

### Peptide synthesis and characterization

P(122-131) (KKKKKEGKKQ) and B(122-131) [Biot-G4-P(122-131)] peptides were produced as previously described, having no cytotoxic effect [29]. The control peptide (5K) (KKKKK) was purchased from Sigma-Aldrich (Saint-Quentin Fallavier, France).

### Adhesion assay

24-well culture plates were coated with 10 μg/ml fibronectin for 1 h at 37°C. Wells were then incubated with a 0.5% solution of bovine serum albumin (BSA) for 1 h at 37°C to block further non specific adsorption of protein. 50.000 resuspended cells in RPMI-1640 medium supplemented with 2.5% FBS were then seeded. After a 10-min incubation period unattached cells were removed by shaking the plates at 2.000 rpm for 10 sec, and by three washes with PBS. Attached cells were fixed with 4% paraformaldehyde and stained with crystal violet.

### Crystal Violet assay

Adherent cells were fixed with methanol and stained with 0.5% crystal violet in 20% methanol for 20 min. After gentle rinsing with water, the retained dye was extracted with 30% acetic acid, and the absorbance was measured at 590 nm.

### Soft agar growth assay

Anchorage-independent growth was assessed by measuring the formation of colonies in soft agar. Twelve-well plates were layered with bottom agar, which consisted of growth medium containing 10% FBS and 0.8% agar. After the bottom agar had solidified, 2000 cells were resuspended in growth medium containing 10% FBS, 0.3% agar, and peptide, then seeded onto the bottom agar. The top agar was then allowed to solidify, and standard growth media supplemented with peptide was added to each well. The cells were incubated at 37°C, in 5% CO_2 _for 12 days. Cell colonies larger than 50 μm were quantified by counting the entire area of each well.

### Transwell assay

Migration assays were performed in Boyden chambers using filters (8 μm pore size, Costar, Avon, France) coated with fibronectin (7,5 μg/cm^2^) for 1 h at 37°C. Filters were washed, blocked with 0.5% BSA for 1 h at 37°C, and dried. Assay medium (RPMI-1640 medium supplemented with 2.5% FBS, and 0.5% BSA, with or without the chemo attractant) was added to the lower compartment, and 10^4 ^cells were added into the insert. After incubation for 30 min at 37°C, filters were fixed. Non-migrated cells were scrapped off the upper side of the filter, and filters were stained with crystal violet. Number of migrated cells was quantified by counting the entire area of the filter.

### Chicken embryo chorioallantoic membrane (CAM) assay

The *in vivo *CAM angiogenesis model was used as previously detailed [10].

### Immunofluorescence confocal microscopy

DU145 cells grown in 8-well tissue culture slides (Nunc) were incubated with 100 μΜ biotinylated P(122-131) (B(122-131)) or with pleiotrophin at 4°C for the indicated time. The cells were then fixed in 4% paraformaldehyde for 10 min at room temperature, rinsed three times with PBS, quenched with 50 mM Tris buffer pH 8.0 and 100 mM NaCl, permeabilized for 15 min in PBS containing 0.3% Triton X-100 and 0.5% bovine serum albumin (BSA), and blocked in PBS containing 3% BSA for 1 h at room temperature. Cells were incubated for 1 h with streptavidin-FITC (1:100), anti-RPTPβ/ζ antibody (1:100), and rhodamine-conjugated goat anti-mouse IgG (1:600) in permeabilization buffer. After three rinses in PBS, cells were mounted using Sigma mounting fluid. Labelling was observed using a Nikon confocal microscope and photographed.

### Reverse transcriptase-polymerase chain reaction (RT-PCR) for RPTPβ/ζ, pleiotrophin, and GAPDH

Total RNA was extracted using the Nucleospin RNA II kit (Macherey-Nagel, Germany), according to the manufacturer's instructions. The integrity of he isolated RNA was examined by electrophoresis on a 1% agarose gel containing 0.5 mg/ml ethidium bromide. Specific primers were as follows: hRPTPβ/ζ, 5"-TTCTGTGCTCTGACAACCCTTA-3" and 5"-AGGAAGAGGAAAACAATGCTCA-3"; hpleiotrophin, 5"-GAGCGCCAGAGAGGACGTTT-3" and 5"-TCCTGTTTGCTGATGTCCTTTT-3" hGAPDH, 5"-CCACCCATGGCAAATTCCATGGCA-3" and 5"-TCTAGACGGCAGGTCAGGTCCACC-3". The RT-PCR reactions were performed in a single step with 250 ng of total RNA, using the Qiagen RT-PCR system. The RT-PCR products were subjected to electrophoresis on 1% agarose gel containing 0.5 mg/ml ethidium bromide, digitally photographed, and quantified using image analysis software (Scion Image PC, Scion Corporation, Frederick, MD).

### Pleiotrophin-antisense RNA transfection

Stable transfection of DU145 to down-regulate pleiotrophin expression was performed as previously described [23]. Briefly, full length cDNA for pleiotrophin was subcloned at EcoRI site in pcDNA3.1+ expression vector (In Vitrogen, Cergy Pontoise, France) in antisense orientation. DU145 cells were seeded in RPMI-1640 medium supplemented with 10% FBS. 24 h later, cells were transfected with the Transfast™ Reagent (Promega Corperation) according to the manufacturer's instructions. The ratio of Transfast™ Reagent to DNA was 2:1. After 1 month of selection with 300 μg/ml G418, clones were screened for down-regulation of pleiotrophin expression. The pcDNA3.1+ expression vector was used as a negative control.

### siRNA transfection

RNA oligonucleotide primers and the siPORT NeoFX Transfection Agent were obtained from Ambion Inc. The following sequences were used: siRNA1 RPTPβ/ζ sense, 5"-AAAUGCGAAUCCUAAAGCGUU-3"; siRNA1 RPTPβ/ζ antisense, 5"-AACGCUUUAGGAUUCGCAUUU-3", siRNA2 RPTPβ/ζ sense 5"-GCGACCAACUGAUUGUCGGA-3"; siRNA2 RPTPβ/ζ antisense, 5"-UCGACAAUCAGUUGGUCGC-3". The annealing of the primers and the transfection was performed according to Ambion's instructions. Briefly, siPORT NeoFX and siRNA were mixed at a final ratio of 1:10 in OPTI-MEM media. The transfection complexes were then overlaid onto 6-well plate cultures grown in RPMI-1640 supplemented with 10% FBS. Transfection efficiency was evaluated using Silencer FAM Labelled GAPDH siRNA (Ambion). Negative control siRNAs from Ambion was also used.

### shRNA transfection

Stable transfection of DU145 cells using shRNA targeting RPTPβ/ζ expression was performed using the pSilencer 4.1-CMV expression vector and the siPORT XP-1 Transfection Agent obtained from Ambion Inc. Based on the siRNA sequence, shRNA was designed, ligated into the pSilencer 4.1-CMV expression vector and transfected into cells according to Ambion's instructions. Briefly, siPORT XP-1 and shRNA were mixed at a final ratio of 1:6 in OPTI-MEM media. The transfection complexes were then overlaid onto 24-well plate cultures grown in RPMI-1640 supplemented with 10% FBS. After 1 month of selection with 300 μg/ml G418, clones were screened for down-regulation of RPTPβ/ζ expression. Double-stranded negative control shRNA from Ambion was also used.

### Immunoprecipitation

Media from DU145 cultures grown in 60 mm plastic dishes were aspirated, cells were washed twice with ice-cold PBS, and cells were lysed in 1 ml buffer containing 50 mM HEPES pH 7.0, 150 mM NaCl, 10 mM EDTA, 1% Triton X-100, 1% Nonidet P-40, 1 mM phenylmethylsulfonyl fluoride, 1 mM sodium orthovanadate, 5 μg/ml aprotinin, and 5 μg/ml leupeptin. Cells were harvested, sonicated for 4 min on ice, and centrifuged at 20.000 g for 10 min at 4°C. Approximately 400 μg of the supernatant was then incubated with 30 μl of protein A-Sepharose bead suspension for 60 min at room temperature. Beads were collected by centrifugation, and the supernatants were incubated overnight at 4°C with anti-RPTPβ/ζ (1:200) or anti-Src (1:1000) primary antibodies. The mixtures were then incubated with 80 μl protein A-Sepharose beads for 3 h at 4°C. The beads and bound proteins were collected by centrifugation (10.000 g, 4°C), washed three times with ice-cold lysis buffer, and resuspended in 60 μl 2× SDS loading buffer (100 mM Tris-HCl pH 6.8, 4% SDS, 0.2% bromphenol blue, 20% glycerol, 0.1 M dithiothreitol). Samples were then heated to 95-100°C for 5 min and centrifuged. Fifty microliters of the supernatant were analyzed by Western blotting.

### Western blot analysis

Cells were starved for 4 h, then incubated with P(122-131) for varying times. Cells were subsequently washed twice with PBS and lysed in 250 μl 2× SDS loading buffer under reducing conditions. Proteins were separated by SDS-PAGE and transferred to an Immobilon-P membrane for 3 h in 48 mM Tris pH 8.3, 39 mM glycine, 0.037% SDS, and 20% methanol. The membrane was blocked in TBS containing 5% non-fat milk and 0.1% Tween 20 for 1 h at 37°C. Membranes were then probed with primary antibody overnight at 4°C under continuous agitation. Anti-RPTPβ/ζ antibody was used at a 1:500 dilution. All other antibodies were used at a 1:1000 dilution. The blot was then incubated with the appropriate secondary antibody coupled to horseradish peroxidase, and bands were detected with the ChemiLucent Detection System Kit (Chemicon International Inc., CA), according to the manufacturer's instructions. Where indicated, blots were stripped in buffer containing 62.5 mM Tris HCl pH 6.8, 2% SDS, 100 mM 2-mercaptoethanol for 30 min at 50°C and reprobed. Quantitative estimation of band size and intensity was performed through analysis of digital images using the ImagePC image analysis software (Scion Corporation, Frederick, MD).

### Statistical analysis

Comparisons of the mean values among groups was performed by means of ANOVA and unpaired Student t-test. Homogeneity of variances was tested by Levene's test. Each experiment included at least triplicate measurements for each condition tested. All results are expressed as mean ± SE. from at least three independent experiments. Values of p less than 0.05 were accepted as significant (*p < 0.05, **p < 0.01, ***p < 0.001).

## Competing interests

The authors declare that they have no competing interests.

## Authors' contributions

All authors participated in the design of the study. ZD performed all experiments and drafted the manuscript. All authors read, revised, and approved the final manuscript.

## Supplementary Material

Additional file 1 **A) Antisense RNA-mediated pleiotrophin knockdown in DU145 cells**. Western blot analysis (WB) and RT-PCR analysis for pleiotrophin. DU145-NC1 and DU145-NC3 cells transfected with pcDNA3.1+ plasmid. DU145-HM2 and DU145-HM5 cells transfected with pleiotrophin antisense RNA. **(B) siRNA-mediated RPTPβ/ζ knockdown in DU145-HM2 cells**. Western blot analysis (WB) and RT-PCR analysis for pleiotrophin and RPTPβ/ζ. siRNA NC siRNA negative control sequence that do not target any known mRNA. Click here for file
