# Supplementary figures and images for: A Pleiotrophin C-terminus peptide induces anti-cancer effects through RPTPβ/ζ
Source: Mol Cancer. 2010 Aug 25;9:224. doi: 10.1186/1476-4598-9-224 (PMC2936342; doi:10.1186/1476-4598-9-224)

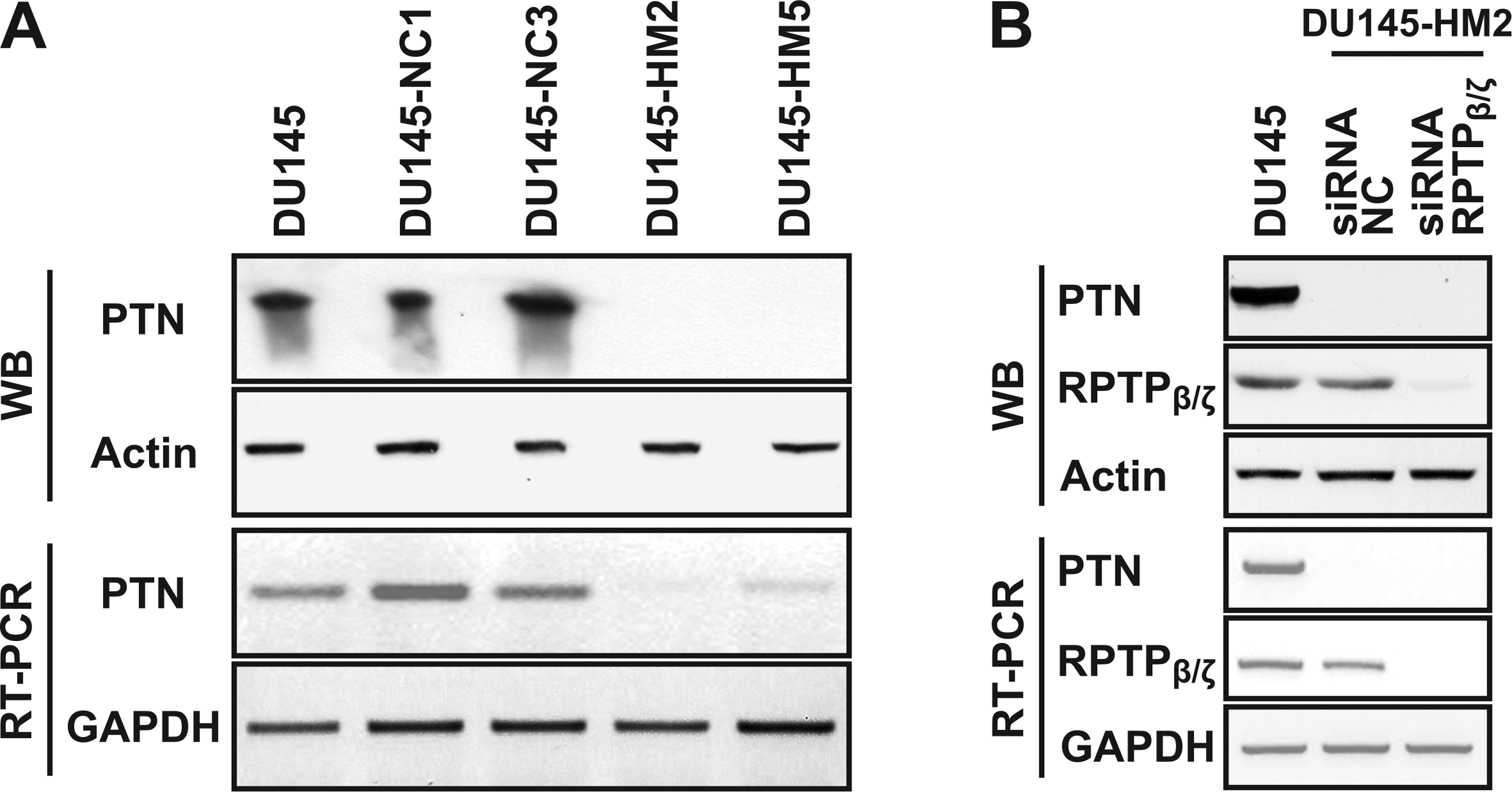

Supplement: Additional file 1 — A) Antisense RNA-mediated pleiotrophin knockdown in DU145 cells. Western blot analysis (WB) and RT-PCR analysis for pleiotrophin. DU145-NC1 and DU145-NC3 cells transfected with pcDNA3.1+ plasmid. DU145-HM2 and DU145-HM5 cells transfected with pleiotrophin antisense RNA. (B) siRNA-mediated RPTPβ/ζ knockdown in DU145-HM2 cells. Western blot analysis (WB) and RT-PCR analysis for pleiotrophin and RPTPβ/ζ. siRNA NC siRNA negative control sequence that do not target any known mRNA. [file 1476-4598-9-224-S1.TIFF]
